# Supplementary material for: The Rasch Analysis Shows Poor Construct Validity and Low Reliability of the Quebec User Evaluation of Satisfaction with Assistive Technology 2.0 (QUEST 2.0) Questionnaire
Source: Int J Environ Res Public Health. 2023 Jan 6;20(2):1036. doi: 10.3390/ijerph20021036 (PMC9859407; doi:10.3390/ijerph20021036)
Supplement: Supplementary file 1 [file ijerph-20-01036-s001.zip › IJERPH QUEST suppl mat 1 2022 11 15.pdf]

SUPPLEMENTAL MATERIALS File S1

Categories functioning: missing responses

1939 data points from 250 questionnaires (111 participants) were fitted to the Many-Facet Rating Scale model. The dataset contained 61 missing responses, corresponding to about 3% of the size of the expected data matrix (2000 data points).

In particular, 221 questionnaires had no missing data, 15 had one unanswered item, and the remaining 14 had blanks in two or more items. At most, there were five missing items in a questionnaire, which happened with a participant only.

The number of missing responses per item is given in the table below.

**Item 1**	**Item 2**	**Item 3**	**Item 4**	**Item 5**	**Item 6**	**Item 7**	**Item 8**
0	13	12	9	9	7	5	6

Differential Item Functioning of the QUEST 2.0 – device

Items	Adult		Elderly		Contrast	S.E.	P-value
	Calibration	S.E.	Calibration	S.E.			
1, dimensions	-0.17	0.15	0.18	0.10	-0.35	0.18	0.048
2, weight	0.29	0.13	0.40	0.09	-0.11	0.16	0.477
3, ease in adjusting	0.27	0.13	0.07	0.10	0.20	0.17	0.232
4, safe and secure	-0.11	0.15	-0.44	0.13	0.33	0.19	0.094
5, durability	-0.44	0.17	-0.36	0.12	-0.08	0.21	0.701
6, easy to use	0.03	0.14	0.12	0.10	-0.09	0.17	0.594
7, comfortable	0.15	0.13	0.06	0.10	0.09	0.17	0.591
8, effective	0.00	0.14	-0.10	0.11	0.09	0.18	0.598

Table S1. Differential Item Functioning of the QUEST 2.0 – Device: age. Respondents were classified as adults or elderly according to their age. Items: item number and keyword. Calibration: item calibration in the respondent group. S.E.: standard error. Contrast: item's calibration difference in the two groups. P-value: the probability that DIF contrast is a random accident (null hypothesis: *"item calibration is the same in the two participants' groups"*).

Items	Males		Females		Contrast	S.E.	P-value
	Calibration	S.E.	Calibration	S.E.			
1, dimensions	-0.05	0.13	0.15	0.11	-0.20	0.17	0.230
2, weight	0.24	0.12	0.46	0.10	-0.22	0.15	0.151
3, ease in adjusting	0.21	0.12	0.09	0.11	0.12	0.16	0.465
4, safe and secure	-0.34	0.14	-0.29	0.13	-0.05	0.19	0.815
5, durability	-0.37	0.15	-0.40	0.13	0.02	0.20	0.914
6, easy to use	0.16	0.12	0.03	0.11	0.12	0.16	0.448
7, comfortable	0.21	0.12	-0.01	0.11	0.22	0.16	0.177
8, effective	-0.06	0.13	-0.06	0.12	0.00	0.17	0.991

Table S2. Differential Item Functioning of the QUEST 2.0 – Device: gender.

Respondents were classified as males or females. Same abbreviations as the previous table.

Items	Patients		Caregivers		Contrast	S.E.	P-value
	Calibration	S.E.	Calibration	S.E.			
1, dimensions	-0.03	0.10	0.24	0.14	-0.27	0.17	0.115
2, weight	0.35	0.09	0.40	0.13	-0.05	0.16	0.751
**3, ease in adjusting**	0.30	0.09	-0.22	0.16	**0.52**	0.19	**0.006**
4, safe and secure	-0.24	0.11	-0.45	0.18	0.21	0.21	0.321
5, durability	-0.37	0.12	-0.42	0.17	0.05	0.21	0.818
6, easy to use	0.04	0.10	0.18	0.14	-0.14	0.17	0.415
7, comfortable	0.01	0.10	0.26	0.14	-0.25	0.17	0.145
8, effective	-0.04	0.10	-0.11	0.15	0.07	0.18	0.701

Table S3. Differential Item Functioning of the QUEST 2.0 – Device: respondent class.

Respondents were patients or caregivers. Same abbreviations as the previous table. Item 3, "ease in adjusting" (bold), showed a large (contrast > 0.5 logits) and significant DIF ($p < 0.01$).

Details of the Rasch analysis iteration

The table below reports additional details on the Rasch analysis (Many-Facet Rating Scale model). It is taken from the recently published RULER guidelines [1,2] to help disseminate Rasch analysis results. The original table has been modified to make it suitable to report results from a Many-Facet analysis.

Analysis	Items	Rating scale categories	Person mean (SD) logits	(*) RMSE	Floor effect n (%)	Ceiling effect n (%)	(**) Global Pearson chi-squared (p-value)	(*) PSR	(*) PSI	(*) Strata	Items with disordered thresholds (n)	Misfitting items (n)	(§) PCAR Eigenvalue 1st PC (%)	(§§) Misfitting persons n (%)
111 participants (250 quesitonnaires), all eight items, original 5 categories structure, unanchored	8	1 to 5	1.90 (1.47)	0.79	15 (16%)	0 (0%)	1690.7 (0.356)	0.71	1.57	2.42	0	0	1.78 (10.2%)	9 (8.11%)

Abbreviations. RMSE: Root Mean Square error; PSR: Person Separation Reliability; PSI: Person Separation Index; PCAR: Principal Components Analysis of the model's Residuals; PC: principal component. (*): sample values, with extremes; (**): global Pearson chi-squared (with p-value), as per Facets output; (§): PCA of residuals was calculated on the sub-sample of participants who were users of mobility assistive devices. Winsteps was used for this analysis; (§§): participants with infit mean square > 1.5 and infit z-standardised value > 1.96

References

1. Mallinson T, Kozlowski AJ, Johnston MV, Weaver J, Terhorst L, Grampurohit N, Juengst S, Ehrlich-Jones L, Heinemann AW, Melvin J, et al. Rasch Reporting Guideline for Rehabilitation Research (RULER): the RULER Statement. Arch Phys Med Rehabil. 2022 July;103:1477–1486.

2. Van de Winckel A, Kozlowski AJ, Johnston MV, Weaver J, Grampurohit N, Terhorst L, Juengst S, Ehrlich-Jones L, Heinemann AW, Melvin J, et al. Reporting Guideline for RULER: Rasch Reporting Guideline for Rehabilitation Research: Explanation and Elaboration. Arch Phys Med Rehabil. 2022 July;103:1487–1498.
