# Supplementary material for: The Rasch Analysis Shows Poor Construct Validity and Low Reliability of the Quebec User Evaluation of Satisfaction with Assistive Technology 2.0 (QUEST 2.0) Questionnaire
Source: Int J Environ Res Public Health. 2023 Jan 6;20(2):1036. doi: 10.3390/ijerph20021036 (PMC9859407; doi:10.3390/ijerph20021036)
Supplement: Supplementary file 1 [file ijerph-20-01036-s001.zip › IJERPH QUEST suppl mat 2 2022 11 15.pdf]

SUPPLEMENTAL MATERIALS File S2

Differential functioning of the QUEST 2.0 – device

The Many-Facet Rating Scale model was used for the Rasch analysis of the QUEST 2.0 – device questionnaire. The following three facets model (participants, items and device classes) was tested:

$$ln\left(\frac{P_{\mathrm{nikj}}}{P_{\mathrm{ni(k-1)j}}}\right) = b_n - (d_i + t_k) - s_j \qquad [1]$$

Equation [1] is read as: *"the natural logarithm (ln) of the ratio between the probability (P) of observing category k for person n answering item i on occasion j and the probability of observing category k – 1 (again for n answering i on j) is equal to the difference between the ability of the person n (b_n) and the difficulty of item i (d_i), the difficulty of being observed in category k relative to category k-1 (t_k) and the difficulty of occasion j (s_j)".*

When applied to the current study, equation [1] becomes: *"the natural logarithm of the ratio … is equal to the difference between the satisfaction level of participant n (b_n) and the satisfaction level required to endorse item i (d_i), the satisfaction level required to score category k (t_k) and the satisfaction level required to endorse device j (s_j)".*

It is important to stress that, as the original Rasch model for analysing dichotomous items and the Rating Scale model and Partial Credit models, the Many-Facet Rating Scale model also *calibrates thresholds*. The Rating Scale and Partial Credit models give these thresholds to the item calibration. With the Many-Facet model, the thresholds are given to the item calibration and the calibration of the third facet. Therefore, the term to the equal sign left in [1] can be read as: *"the probability of passing the (Andrich) threshold…".*

In the Rasch measurement theory, the person's ability is marked by the threshold with a fifty-fifty probability of passing and failing.

Hence:

$$ln\left(\frac{P_{\mathrm{nikj}}}{P_{\mathrm{ni(k-1)j}}}\right) = b_n - (d_i + t_k) - s_j = 0$$

And:

$$b_n = d_i + t_k + s_j \qquad [2]$$

The ability of person n is given by the sum of difficulty of item i, threshold k and occasion j when person n has a 50% chance to pass the threshold identified by i, k and j.

Figure 1 shows the map of QUEST 2.0 – device. The leftmost and rightmost vertical panels show the line of the variable: they report the satisfaction measures in logit from low (negative values, lower part) to high (positive values, higher part). The three middle panels report, from left to right, the calibration of devices, items, and categories. 0 logits (*, horizontal line) report the mean facet calibration. Dashed lines (---) in the categories facet mark the half-score thresholds, the transition point into one score from the one below. The output from Facets 3.84.0 software is shown, with slight modifications. For graphical reasons, the calibration of items 6, 7 and 8 has been omitted since they are the same as items 1 and 3.

Imagine that a prosthesis user scored 3 on item 2 "weight". We are interested in knowing their satisfaction measure.

The participant could have scored 3 because their ability corresponds precisely to the 2-3 half-score threshold. Given a sample of persons whose measure corresponds to the 2-3 threshold, these persons would answer category 2 or 3 on item 2 so that, on average, the sample mean category would be 2.5. For a person with this measure, the chance to pass equals that of failing the threshold. Of course, the participant could have scored 3 even when their ability corresponds to the 3-4 threshold. In this case, the mean category of the sample of respondents would be 3.5. On these bases, it is a reasonable conclusion that the participant's ability lies somewhere between the 2-3 and 3-4 thresholds.

This range of patient measures can be found easily by sliding the maps, as shown in Figure 2.

The middle (*) of the items facet is aligned to the prostheses' location on the measures line (leftmost red segment). Next, the centre (*) of the categories facet is aligned to calibration of item 2 "weight" (rightmost red segment). Lastly, the position of the 2-3 and 3-4 thresholds is found on the measure lines (red dashed segments). In this example, the respondent measure is about 1.2 to 1.8 logits.

The three vertical green segments mark the vertical displacements of the items and categories facets and the further upward displacement of the 3-4 threshold. Note that the green segments correspond (left to right) to s_j (the prostheses calibration), d_i (the item 2 calibration) and t_k (the calibration of the 3-4 threshold). A similar argument applies to the 2-3 threshold. Figure 2 shows that equation [2] can be used in geometric terms by sliding the questionnaires maps.

Imagine now that we are interested in knowing the satisfaction measure of a communication aid user that also scored 3 on item 2 "weight". By appropriately sliding the maps, it is found that their measure is about -1.2 to -0.6 logits (Figure 3).

The two participants' scores on item 2 are the same (3), but their measures differ: scores function differently in users of different devices. Comparing the QUEST 2.0 – device total raw scores of users of various assistive devices could thus lead to wrong conclusions. On these bases, it has been proposed in the main text – Discussion that different devices with different calibrations can indicate differential functioning of the questionnaire.

We presented the case of two persons scoring the same but with different satisfaction measures. But the reverse is true as well. Two persons, users of different devices with different scores on the same item, could measure the same. For example, a communication aid user scoring 4 on item 2 has an average measure of about 0 logits, similarly to a user of seating aids scoring 3 on this item. Again, it is enough to slide the maps to check this.

The Many-Facet model was developed in education to rule out the raters' bias from student evaluations eventually.

In a schooling scenario, the last example would be as follows. Students A and B scored 4 and 3 on test 2, respectively. However, even if their score is different, their (say) mathematical knowledge level is the same. Their score is different because they were assessed by two teachers with different severity/leniency. Rater "communication aid" is more lenient than rater "seating aids" (or, from the opposite point of view, "seating aids" is more severe than "communication aids"). The Many-Facet model represents a formal solution to this problem, making it possible to have unbiased measures.

Applying this idea straight to the medical field, as done in the current study, could be hard to accept from a clinical point of view. The following vignette clarifies this point.

A clinician could say: *"but why should I consider a prosthesis user scoring 1 on item 2 "weight" happy with their device the same as a communication aid user scoring 4 on the same item? The prosthesis user tells me they are "not satisfied at all" with the prosthesis weight. The communication aids user says instead that they are "quite satisfied" with the device's weight".*

In line with the above, the psychometricians' answer would be simply pointing out that this happens because the "weight" of a prosthesis is much more difficult to endorse than that of communication aids. The point is overt if seen from a different perspective.

Consider two services, A and B, each assessing patients and providing assistive devices. An agency financially supports the two services and uses different indicators to monitor the two services. Since patient satisfaction is an independent indicator of care quality[1–3], the agency also asks the services to administer the QUEST 2.0 – device to their clients.

On median, the assistive device users of service A scored 38 on the QUEST 2.0 – device, while those of service B scored 26. The agency would conclude that device users are more satisfied with service A than B and start thinking that service A works better than B. But is this a fair conclusion?

Service B specialises in lower limb amputation and mainly provides prostheses to these patients. On the contrary, service A has a large sample of persons with extreme mobility impairment because of lateral amyotrophic sclerosis and specialises in communication aid provision.

The current Rasch analysis of the QUEST 2.0 – device with the Many Facet model highlights that the agency's conclusion wouldn't be fair. QUEST 2.0 – device score is lower for service B because the device map shows that prostheses are more difficult to endorse. Rasch measures from the Many Facet model should be used instead of ordinal scores to compare the two services fairly.

Figure S1: QUEST 2.0 – device map.

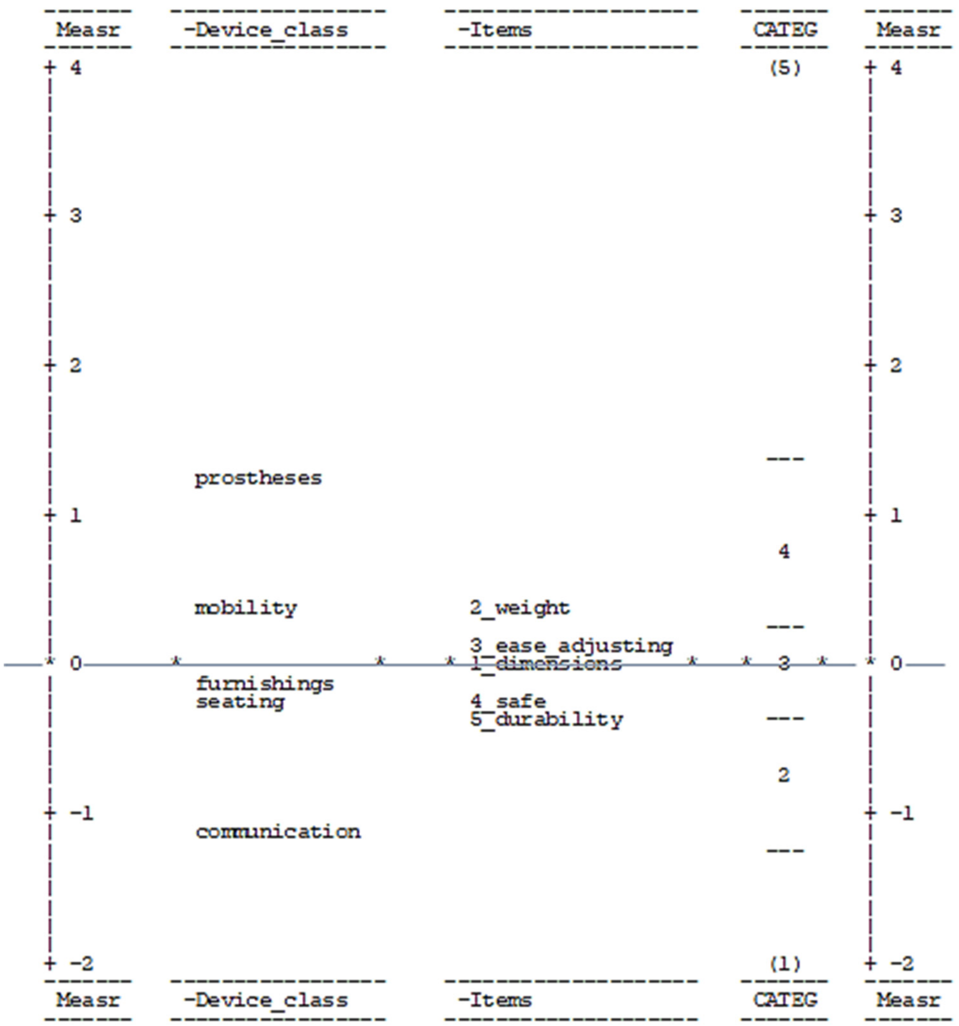

See text for a figure explanation.

Figure S2: use of the questionnaire map for person measurement.

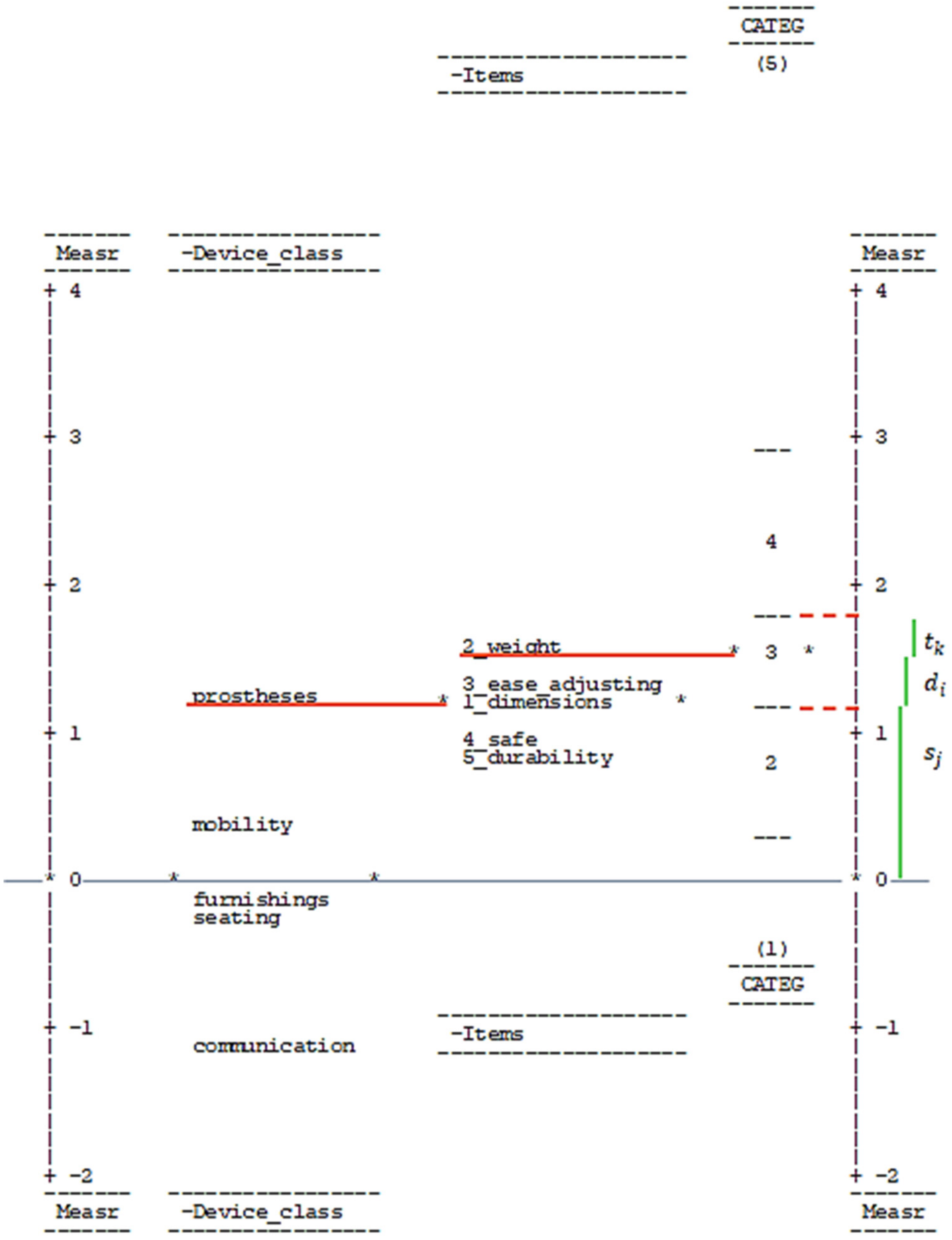

A prosthesis user scored 3 on item 2 "weight". Their measure is about 1.2 to 1.8 logits (range of measures, Measr, between the two red dashed segments).

Figure S3

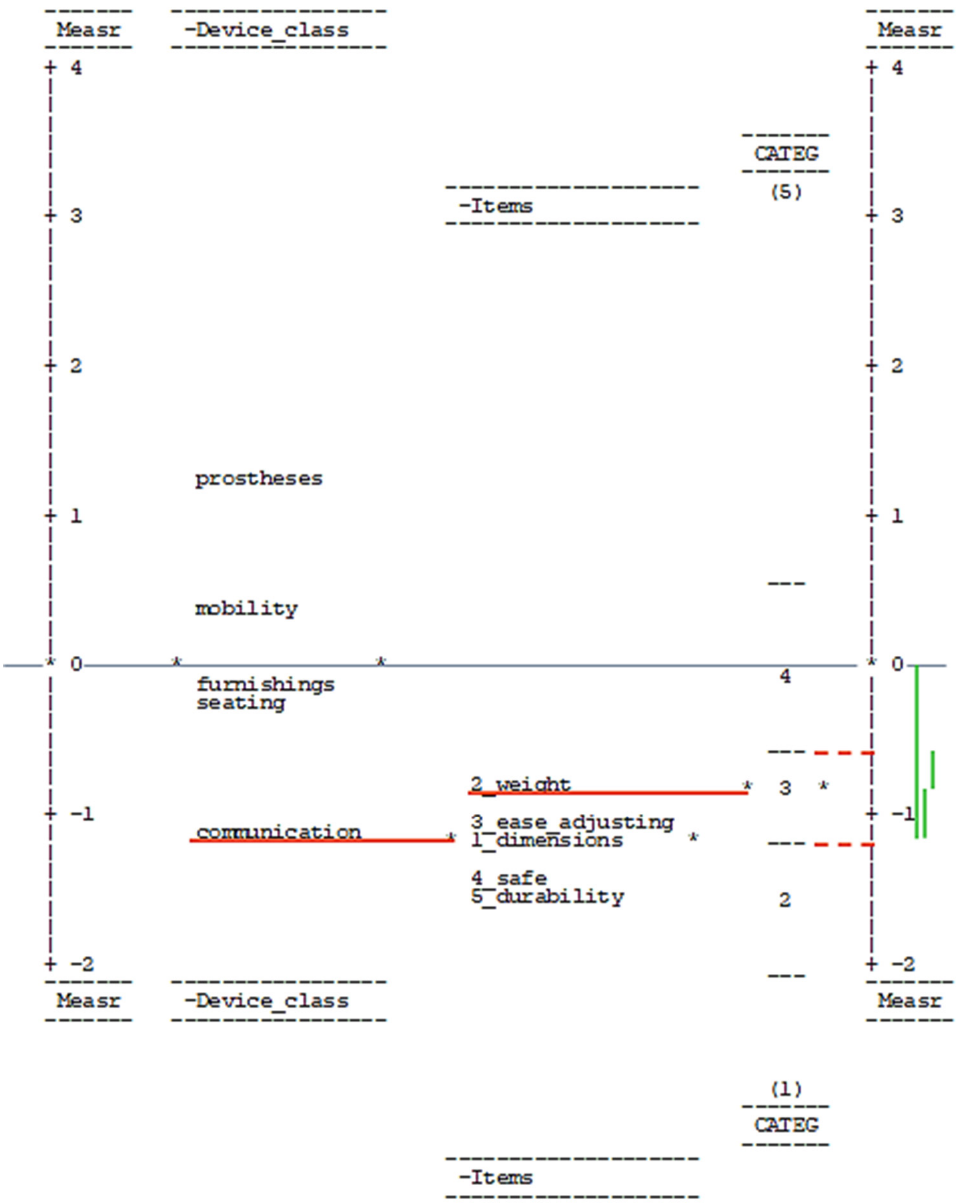

A communication aid user scored 3 on item 2 "weight". Their measure is about -1.2 to -0.6 logits.

References

1. O'Connell B, Young J, Twigg D. Patient satisfaction with nursing care: a measurement conundrum. Int J Nurs Pract. 1999;5:72–7.

2. Franchignoni F, Ottonello M, Benevolo E, Tesio L. Satisfaction with hospital rehabilitation: is it related to life satisfaction, functional status, age or education? J Rehabil Med. 2002;34:105–8.

3. Cimas M, Ayala A, García-Pérez S, Sarria-Santamera A, Forjaz MJ. The patient satisfaction questionnaire of EUprimecare project: measurement properties. Int J Qual Health Care. 2016;28:275–80.
